# Evaluating Socratic inquiry, reflection and argumentation as strategies for critical thinking

**DOI:** 10.4102/curationis.v48i1.2691

**Published:** 2025-09-17

**Authors:** Glen T. Malape, Agnes Makhene, Eunice Mutava

**Affiliations:** 1 Nursing Science Department, School of Health Care Sciences, Sefako Makgatho Health Sciences University, Pretoria, South Africa; 2 Department of Nursing, Faculty of Health Sciences, University of Johannesburg, Johannesburg, South Africa

**Keywords:** socratic inquiry, reflection, argumentation, implementation, evaluation, facilitate, critical thinking, nursing education

## Abstract

**Background:**

Rapid technological advancements, the ageing population and the complex disease management processes significantly change the healthcare environment, fostering a dire need for nurses who can think critically. Critical thinking (CT) is foundational to clinical competency. However, there is a shortage of evidence on the identification of the most suitable pedagogies to promote CT.

**Objectives:**

This article aims to implement and evaluate the effectiveness of Socratic inquiry, reflection and argumentation as CT facilitation strategies in nursing education.

**Method:**

A quantitative, quasi-experimental, pretest-post-test research design was used. Thirty-two student nurses were selected through convenience sampling. Data were gathered using the Holistic Critical Thinking Scoring Rubric (HCTSR) and analysed through descriptive and inferential statistics, using the SPSS software version 28.0.

**Results:**

The results showed that the three strategies were successful in promoting CT. Overall, Socratic inquiry, reflection and argumentation were effective in enhancing students’ CT; five of the six domains of CT saw a statistically significant improvement following the implementation of the three facilitation strategies.

**Conclusion:**

Socratic inquiry, reflection and argumentation are effective pedagogies for the facilitation of nursing students’ CT. Evidenced by improved post-test means of all CT attributes and statistically significant improvements in five of the six CT competencies.

**Contribution:**

The findings of this study demonstrate the effectiveness of Socratic inquiry, reflection and argumentation in enhancing nursing students’ CT. The study also makes recommendations on the improvement of nursing practice, education and policy development, to foster a system that will create nurses who can think critically to enhance patient outcomes.

## Introduction

Critical thinking (CT) refers to one’s capacity to analyse information, assess its relevance, make reasonable judgements as well as appropriately interpret and apply it as a problem-solving technique (Fitri et al. 2025). It is defined as a:

[*P*]urposeful, self-regulating judgment that results in interpretation, analysis, evaluation and conclusion as well as an explanation of the evidential, conceptual, methodological, critical or contextual considerations on which that judgment is based (Facione 1990).

The ability to think critically is essential to nursing because it provides nursing students with higher-order thinking skills, which enable them to formulate appropriate answers to problems and to act quickly (Tseng et al. 2022; Zhang & Chen 2021).

It is a mental activity that can be regularly practised and improved in the field of nursing. A nurse is unable to identify the patient’s problem due to the lack of CT skills. As nurses choose treatment methods and evaluate acquired data, CT is necessary to make quick and informed judgements (Abiogu et al. 2020). In order to execute safe care practices and respond appropriately and suitably to society’s health needs, nurses must be able to gather and critically evaluate information as well as think critically about it in a logical and objective approach (Butler 2020; Grecu 2023).

There are numerous points of view about what constitutes CT (Ma, Zhang & Luo 2021). Some make it as simple as calling CT excellent or superior thinking. Despite prolific publications on CT in nursing education, there is still an evidence gap on suitable facilitation strategies to promote this crucial skill (Towfik et al. 2023). As such, this article will focus on the implementation and evaluation of three facilitation strategies. The strategies included Socratic inquiry, reflection and argumentation. Socratic Inquiry describes a dialogic facilitation technique that uses questions and reflections to encourage investigation and CT (Williams & Zimmerman 2022) whereas reflection is a method in which one interacts with one’s thoughts, feelings and actions as well as the abstract basis that underlies them to gain insights into the process of change (Yang et al. 2022). The other pedagogical strategy is argumentation, which refers to a conversational, debate-based and logical activity to show that one or more arguments are persuasive by providing evidence to support or contradict the proposals (Meral, Şahin & Akbaş 2021).

### Study aim

This study aimed to implement and evaluate the effectiveness of Socratic inquiry, reflection and argumentation as CT facilitation strategies in nursing education.

## Research methods and design

In this study, a quantitative, quasi-experimental, one-group pretest-post-test research design was used to implement and evaluate Socratic inquiry, reflection and argumentation as CT facilitation strategies. The CT levels of nursing students were measured twice using the Holistic Critical Thinking Scoring Rubric (HCTSR); data were collected on two occasions, before and after the intervention. The subject matter that was taught was a part of the prescribed curriculum. As such, no students could be excluded from attending the lessons. The study therefore lacked a control group. The quasi-experiment fostered the collection of the students’ baseline CT competencies in the pretest and compared the findings to determine the effectiveness of the intervention with the post-test scores.

### Setting

The research was conducted in a nursing department at a higher education institution (HEI) in the Gauteng province. The province is home to 7 of the 26 public universities in the country. Nursing is one of the 10 departments in the Faculty of Health Sciences at the university, which is one of nine faculties at the university, highlighting the multidisciplinary structure of the university. The department offers the Bachelor of Nursing and Midwifery (R174) nursing curriculum over a 4-year period, on a full-time basis. The university has a broad reach in recruitment, allowing the nursing department to admit students from all nine provinces in the country, also offering postgraduate diplomas in health service management, nursing education and clinical postgraduate diplomas, including occupational health nursing, midwifery and primary care nursing. Furthermore, the department also offers master’s and doctoral degree programmes.

### Population and sampling

The target population were all the student nurses registered for the R174 curriculum at the HEI. A non-probability convenience sampling method was used to select the participants for this study. Therefore, the eligibility criteria included being a third-year nursing student, enrolled in the R174 Bachelor of Nursing and Midwifery programme at a higher education facility in Gauteng province, South Africa. Students who took part in the pilot study and those who were not enrolled in the R174 nursing programme were not eligible to participate in the study and were thus excluded.

Because this study was intended for senior students, the third-year group was selected as the accessible sample. Because of clashing clinical and theory student placements, the fourth-year group was inaccessible during the data-collecting phase. In addition, the module composition and learning content covered in levels 3 and 4 were also different, making it impossible for the intervention to be implemented in both groups. Instead, the third-year group was chosen to undergo the intervention. For this reason, the sample size was 32 following consultations with a statistician from STATKON that deemed it feasible, the researcher proceeded with the study.

### Intervention

The researcher, who was a lecturer at another nursing education institution and registered with the South African Nursing Council (SANC) as a nurse educator, adopted a facilitator role at the selected research site for the purpose of the study. The three strategies were implemented in no particular order as most learning activities comprised all three strategies within one lesson. The researcher developed lesson plans for the general nursing science learning outcomes of the third-year level. The participants were asked to complete learning activities that align with the prescribed curriculum as well as the corresponding learning outcomes, for which the researcher formulated lesson plans.

**FIGURE 1:**
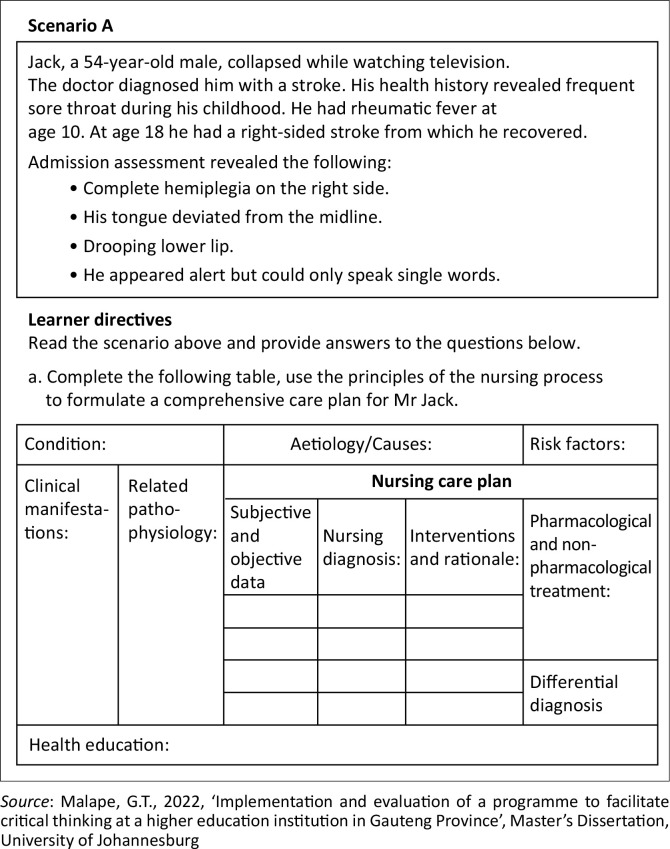
Learning scenario for neurological system disorders: Infectious and inflammatory neurological disorders learning unit.

The lesson plans comprised inquiry-based, reflective and argumentative learning activities, centred around the scientific nursing process, requiring students to design comprehensive nursing care plans based on ill-structured problems provided by the facilitator in the form of a scenario. An example of these scenarios is provided in Figure 1.

**FIGURE 2:**
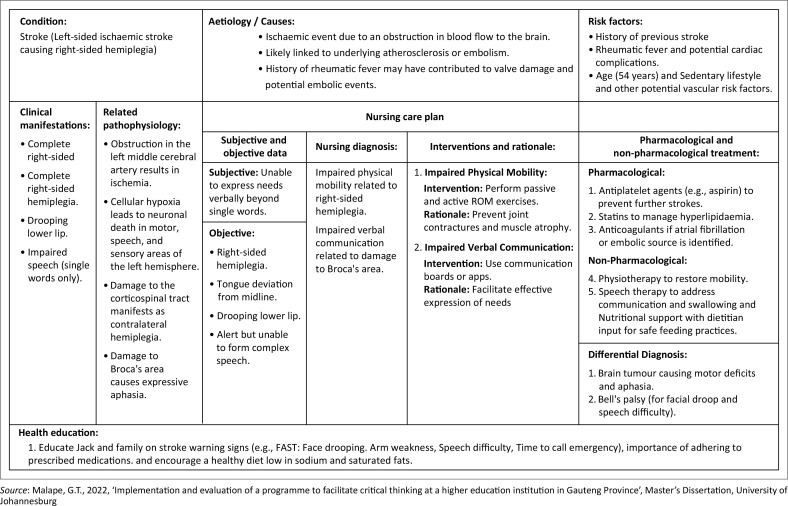
An example of a nursing care plan by the student based on the problem-based scenario.

These scenarios are complex, vague and open-ended problems which mimic real-life situations, with multiple possible solutions (Jaleniauskienė & Jucevičienė 2018). They lack vital elements that could be too leading for the students, making it overly simple for them to solve the problem, thus compromising knowledge creation and CT. Instead, these scenarios required that the students explore the problem in depth for them to be able to formulate suitable care plans. The care plans were focused on specific medical and surgical conditions per body system as set out in the curriculum. For each learning unit, the students presented their care plans to the researcher and the rest of the class (Figure 2).

These were succeeded by reflective exercises where students were tasked to identify shortfalls in their care plans and collaboratively formulate means to bridge the gaps, comparing their answers with those of their peers. Moreover, additional questions to foster debate within the classroom included a critical analysis and description of the credibility, context and relevance of the information gathered by the students in attempting to solve the problem using credible information.

In their responses, they proposed different approaches to solving the problem; the students were required to support their care plans with reflections, justifying the assessment, diagnosis, planning, implementation and evaluation phases of the scientific nursing process. Students were probed to pose inquiries towards what was being presented to them and these were countered by the class in an argumentative manner to further promote discourse. The discourse would lead the students towards probing, which led to further personal reflection, reorganising and debating their stances regarding the care plans they had designed.

The critical discourse allowed the students to collaboratively determine the applicability of their care plans and warranted adjustments and modifications where required, thus allowing further refinement of the care plan, ensuring the best possible nursing care for the patient. The use of ill-defined case scenarios to encourage reflection and the integration of questioning techniques as well as argumentative discourse fostered a comprehensive and collaborative interaction. This allowed students to clarify misconceptions, acknowledge opposing stances or contest them with evidence and adapt their previously skewed views.

### Data collection

The study was conducted over 21 months, from February 2021 to October 2022. Pretest and post-test data were gathered prior to and after the intervention respectively. This was conducted through a researcher-administered scoring rubric, known as the HCTSR. The rubric was composed of demographic data and a four-point Likert scale. The Likert scale was as follows: 1 point was assigned for ‘significantly weak’ CT, 2 points for ‘unacceptable’ CT, 3 points for ‘acceptable’ CT skills and 4 points for ‘strong’ CT. Scores of 3 and 4 on the CT scale show proficient CT, whereas 1 and 2 suggest little or no CT (Chapwanya 2021; Sharadgah, Sa’di & Ahmad 2020).

The HCTSR evaluates CT by measuring six fundamental skills: self-regulation, inference, evaluation, interpretation, analysis and explanation (Facione 1990; Sharadgah et al. 2020). The validity of the HCTSR has been established in previous studies conducted in different contexts, reporting a high Cronbach’s alpha of 0.88 (Facione & Facione 1994), which led to the validity and reliability of the instrument not being assessed for this specific group of students. Nonetheless, a pilot study composed of 18 second-year students was conducted, to determine the instrument’s applicability, usability and contextual relevance. The pilot study indicated that the instrument was fitting for the study, without any flaws and did not require any modifications or enhancements.

The interrater reliability of the HCTSR was previously reported with reasonably high Cohen Kappa scores of 0.71, 0.75 and 0.81 between three evaluators (Sharadgah et al. 2020). As such, it was not recalculated in this study. However, to ensure consistency in scoring, two assessors were trained using the same scoring rubric before the data collection process. Both assessors independently evaluated participants’ CT levels, and discrepancies were discussed and resolved to minimise potential subjectivity (Nieswiadomy & Bailey 2018; Sharadgah et al. 2020). Furthermore, no changes were made to the instrument’s layout or scoring criteria once data collection commenced, and the same standardised scoring process was adhered to throughout the study. These measures ensured uniformity and reliability in evaluating the participants’ CT levels.

### Data analysis

Data analysis was conducted using the Statistical Program for the Social Sciences version 28.0 (SPSS Incorporated, Chicago, Illinois, United States). Data were first logged onto Microsoft Excel (Microsoft Incorporated, Redmond, Washington, United States), which facilitated data organisation. The data were subsequently cleansed to determine correctness and completeness before it was transferred to the SPSS program for analysis using descriptive and inferential statistics (Nieswiadomy & Bailey 2018).

### Ethical considerations

Before the commencement of the study, ethical clearance was obtained from the Faculty of Health Sciences Research Ethics Committee at the University of Johannesburg, (clearance number: REC-1272-2021). In addition, further clearance was obtained from the Higher Degrees Committee (clearance number: HDC-01-67-2021).

Permission was also obtained from the executive director of research and innovation at the university as the gatekeeper to permit the researcher access to the students in their learning environment. Consent was obtained from all the students who participated in the study; they were allocated pseudonyms instead of using their real names or any personal details that had the potential to compromise their anonymity and confidentiality.

The statistician involved in the study also signed a confidentiality agreement. All collected data were stored in encrypted folders in a password-protected computer. Lastly, of the 39 students who made up the sample, 7 autonomously declined to be a part of the study and did not sign informed consent. Their CT scores were therefore not obtained in the pretest and post-test of the study. However, to uphold the principle of justice, they were allowed to attend the lessons without any prejudice as the content that was taught was a part of the prescribed curriculum of the R174 programme they were enrolled in.

## Results

Quantitative data gathered from research participants were sorted and summarised using descriptive statistics. They enabled the researcher to examine the characteristics, behaviours and experiences of the participants (Nieswiadomy & Bailey 2018).

From the selected sample, 32 third-year Bachelor of Nursing and Midwifery students underwent the intervention at the selected HEI. Table 1 shows the demographic data of the sample. Five strata were used to classify the students’ ages. Stratum one was composed of one (3.12%) 18-year-old, who was the youngest of the group. Stratum two consisted of 16 (50.00%) students, ranging between the ages of 19 and 21. The third stratum ranged from age 22 to 25 and was composed of 11 (34.38%) students. Students aged 26 to 27 made up the fourth stratum and they were three (9.38%). In stratum five, there was one (3.12%) student who was the oldest of the entire sample at 28 years of age. The sample was female dominated with 22 (68.75%) female students and 10 (31.25%) male students. Of the 32 students that made up the sample, 30 (93.75%) had no previous higher education history and 2 (6.35%) had a history of previous higher education studies.

**TABLE 1:** Demographic data of the sample.

Variables	Frequency	%
**Age group (years)**		
18	1	3.12
19–21	16	50.00
22–25	11	34.38
26–27	3	9.38
28	1	3.12
**Gender**		
Male	10	31.25
Female	22	68.75
**Previous higher education studies**		
Yes	2	6.25
No	30	93.75

*Source*: Malape, G.T., 2022, ‘Implementation and evaluation of a programme to facilitate critical thinking at a higher education institution in Gauteng Province’, Master’s Dissertation, University of Johannesburg.

### Inferential statistics

Paired sample *t*-tests were conducted to compare the students’ mean pretest and post-test scores.

Table 2 illustrates a statistically significant *p*-value of 0.00 (*p* < 0.05) and a positive strong relationship (*r* = 0.73?) between the pretest and post-test mean scores for overall CT competency.

**TABLE 2:** Pearson pretest-post-test mean correlations.

Test Pair	*N*	Correlation	One-sided *p*
Pair 1 Pretest-post-test means	32	0.73†	0.00

*Source:* Malape, G.T., 2022, ‘Implementation and evaluation of a programme to facilitate critical thinking at a higher education institution in Gauteng Province’, Master’s Dissertation, University of Johannesburg.

† , Correlation is significant at the 0.01 level (2-tailed).

A paired-sample test was conducted to evaluate the impact of the three facilitation strategies on CT scores as shown in Table 3. The statistics showed that the mean post-test score (*M* = 2.79, s.d. [standard deviation] = 0.56) was higher than the mean pretest score (*M* = 2.52, s.d. = 0.65), indicating an improvement in CT after the implementation of the facilitation strategies. The test revealed a statistically significant difference between pretest and post-test scores (< 0.00). The mean difference was 0.28, with a 95% confidence interval ranging from 0.16 to 0.39. The effect size was 0.45, as measured by Cohen’s *d* (Cohen 1988), indicating a moderate practical significance of the strategies on students’ CT.

**TABLE 3:** Paired samples analysis for critical thinking.

Measure	Pretest		Post-test		MD ± s.e.	*t*	*df*	*p*	Effect size (Cohen’s *d*)
	Mean	s.d.	Mean	s.d.					
Critical thinking	2.52	0.65	2.79	0.56	0.28 ± 0.11	−3.87	9	< 0.00	0.45

*Source:* Malape, G.T., 2022, ‘Implementation and evaluation of a programme to facilitate critical thinking at a higher education institution in Gauteng Province’, Master’s Dissertation, University of Johannesburg.

s.d., standard deviation; s.e., standard error; *df*, degrees of freedom; MD, mean difference.

## Discussion

This research aimed to implement and evaluate the efficacy of Socratic inquiry, reflection and argumentation as pedagogies to facilitate CT. The results revealed that these three strategies are generally effective in enhancing CT. The results also showed that the sample was predominantly female, similar to prior research (Lotan 2019) displaying a male underrepresentation as is the case in a South African context with male students making up only 8% of the entire nursing student population (Shakwane 2022). These findings are globally prevalent, owing to the notion that nursing is a female profession (Bordelon et al. 2023, Gauci et al. 2022). As such, men are faced with stereotypical biases and discrimination, questioning of male nurses’ sexual orientation as well as limitations brought about by cultural and religious aspects which deem them unfit to practice nursing. These restrictions are further strengthened by socially influenced gender roles on what is considered a norm thus leading to dysfunctional and uncomfortable work environments (Aynaci & Gulmez 2019; Prosen 2022).

In a quasi-experimental study assessing the impact of an educational intervention, the results showed that women obtained relatively higher CT scores as compared to their male counterparts (López et al. 2020). Essentially because of the belief that women easily take up the caregiver role, because of childhood sociocultural influences, which is a characteristic that is proximally related to CT competency development (Liu et al. 2019). Additionally, previous studies have found age to play a significant role in CT, whereby younger students obtained relatively lower CT scores as compared to older students (Mousazadeh et al. 2021).

However, there being only two age outliers, students aged 18 and 28 rendered the age demographic unreliable as a source of variability. Only 2 of the 32 students had previously undergone higher education experience in this research. As such, the effect of this variable on CT was not assessed. However, previous studies have reported inconsistencies regarding the correlation between age and CT (Demir, French & Hand 2023). For instance, in a study aimed at determining the link between age and CT of students in the health sciences, the results did not demonstrate any statistically significant correlation (Wettstein et al. 2011).

Similarly, prior research has shown that previous educational experience may not only influence the implementation of CT facilitation strategies but may also be a hindrance to the facilitation of CT. As such, a solid link must be established between the conventional approach and traditional facilitation strategies so that students can fully comprehend what they are experiencing (Fernández-Peña et al. 2016). Pedagogically, prior research has primarily focused on strategies that lacked learner-centredness and active student participation, with limited facilitator-student interaction. As such, their approaches fell short of enhancing students’ CT capacity (López et al. 2020). Conversely, the strategies employed in this study foster maximum student participation, allowing them to question themselves and others while constructing their own knowledge. This collaborative approach promoted the sharing of ideas and thoughts, critical analysis of the self, the environment and others thus fostering meaningful learning and promoting CT.

In a scoping review by Westerdahl et al. (2022) the results demonstrated that the most commonly used approaches aimed at enhancing CT were solely facilitative in nature or used in conjunction with a Socratic approach, which was the case in this study. This accentuates the need for efforts that encourage active knowledge construction by students, instead of passive knowledge transmission from the educator to the students. Instead, the facilitator assumed positive and authoritative role-modelling, creating a safe learning sphere that allows the sharing of varying perspectives and aiding the students in shaping their own experiences. The results also showed that the pedagogical strategies employed in this study were effective in enhancing students’ CT overall. These results together with a two-sided *p*-value (*p* < 0.05), indicating statistical significance show that the intervention led to significant improvements in the students’ CT in both measurements. This is depicted in Table 2. Additionally, the results also showed a strong correlation between the scores obtained in the pretest and the post-test. Lastly, there was also a moderate effect size of the three strategies on the students’ CT skills (Table 3).

There is consensus on the significance of CT in higher education although there are still arguments as to how this essential skill is to be effectively facilitated (Bellaera et al. 2021). In this study, CT was facilitated using the infusion approach through which the students were taught CT and the learning content explicitly. Teaching CT along with the subject matter in this manner ensures retention of CT skills by students and also enhances students’ interest, motivation and desire towards the learning content (Orhan & Çeviker-Ay 2023). CT can also be facilitated through other approaches, the general, immersion and mixed approaches (Ennis 1989).

These categories differ based on the explicitness of how CT competencies are facilitated in relation to the learning content (Zhang & Yuan 2022). The generalist method is founded on the notion that CT skills bear interdisciplinary transferability. The immersion approach, similar to the infusion approach is based on the notion that CT is closely linked to domain knowledge, the distinction between the two approaches being that the immersion approach does not constitute explicit and direct teaching of CT skills. The mixed approach, on the other hand, constitutes the general approach with the immersion or the infusion approach (Orhan & Çeviker-Ay 2023; Wynekoop & Nakatani 2023).

Previous empirical results have demonstrated the efficacy of the infusion approach in CT instruction (Bensley & Spero 2014; Taghinezhad & Riasati 2020). Darby and Rashid (2017) also appraised the efficacy of the infusion approach on CT enhancement of engineering students through a quasi-experiment. The experimental group underwent the infusion approach whereas the control group underwent a conventional instruction method. Although the pretest results demonstrated no significant variability of CT skills in both groups, the post-test results demonstrated significantly higher CT skills in the intervention group as compared to the non-experimental group. There exists a close relationship between CT and the nursing process (Westerdahl et al. 2022) thus justifying the use of the nursing process learning content in the context of this study. The existence of this interrelatedness was emphasised by Wilkinson (2011), highlighting that CT is the impetus for the nursing process and that each step of this process constitutes CT. These steps include assessment, diagnosis, planning, implementation and evaluation (Souto et al. 2024).

The use of the nursing process as a problem-solving strategy in CT instruction fosters clinical decision-making (laslan et al. 2023). The results of this study in conjunction with previous empirical findings highlight that using nursing process-based scenarios for CT facilitation fosters theory and practice competency thus ensuring that the populace of students transitioning into registered nurses is adequately prepared to enter the nursing profession. This study also assessed nursing students’ CT competency to determine the differences between the pretest and post-test means. The results demonstrated the efficacy of the three pedagogical strategies employed, through statistically significant improvements overall (Table 3).

Despite the usage of the nursing process, which students start learning and gradually applying from the commencement of the course and continue within the industry, during the implementation phase of the study, students constantly sought clarity on what the facilitator expected of them. They struggled to explain or justify certain stances and there seemed to be a language issue, leading to students asking if they could use vernacular in expressing themselves. This may be attributed to the assertion that less than 10% of South Africans are native English speakers, which warrants a quality enhancement in pedagogical methodologies because the language of teaching is a second language to a large portion of the population (Manditereza 2014).

This observation showed partial consistency with previous studies. A study by Saputri and Rinanto (2018:1–6) evaluated students’ CT skills. The results showed that the students did not perform well in the explanation domain. This suggested that the participants could not strengthen their arguments because they merely offered descriptions that lacked the formation of rational links between concepts. Contrary to what explanation requires, this is the ability to clarify processes or causative relationships thus reinforcing their arguments. Putra, Prayitno and Maridi (2018) speculate that this can result from a lack of confidence that causes one to formulate inaccurate arguments.

### Recommendations

The faculty should provide nurse educators with opportunities for ongoing professional development that supports CT and helps them serve as positive role models for this crucial skill (Raymond et al. 2018). To encourage dissonance among the students and promote knowledge creation, nurse educators should foster a learning environment where student nurses can debate their ideas and competing opinions. Nurse educators should avoid using conventional teaching methods and instead use teaching techniques that promote evidence-based practice and help nursing students recognise problems and pursue solutions. High-fidelity simulation, inquiry-based learning, reflection, research- or problem-based learning, argumentation and flipped classroom are some of these techniques (Dehghanzadeh & Jafaraghaee 2018; Meral et al. 2021; Mthiyane & Habedi 2018; Seibert 2021). Lastly, healthcare organisations should support nurses’ ongoing professional development and enhance their CT, clinical reasoning and clinical judgement to enhance patient care. In order to further support patient outcomes, for future research directions researchers could also conduct longitudinal studies to determine the impact of these strategies over time, particularly following graduation and entry into the profession.

### Limitations of the study

The sampling approach was limited to non-random convenience sampling, lacking a control group because the content that was taught was in the prescribed curriculum.

## Conclusion

The results of this study show the efficacy of Socratic inquiry, reflection and argumentation in facilitating CT. The recommendations for faculty and practice on fostering CT in nursing education and practice are provided. The employment of these pedagogical strategies is vital for knowledge creation as this will result in students and nurses who can think critically, with improved clinical competence and ultimately enhance patient outcomes.
